# Correction: Schwab et al. Spatial Raman Spectroscopy to Characterize (Sulfated) Glycosaminoglycans in Human Articular Cartilage. *Int. J. Mol. Sci.* 2025, *26*, 9875

**DOI:** 10.3390/ijms27115044

**Published:** 2026-06-03

**Authors:** Andrea Schwab, Jannik Jahn, Kerstin Sitte, Christoph H. Lohmann, Jessica Bertrand, Sonja Gamsjaeger

**Affiliations:** 1 Department of Orthopaedics, Medical Faculty, Otto-von-Guericke University, 29120 Magdeburg, Germany; jannik.jahn@med.ovgu.de (J.J.); christoph.lohmann@med.ovgu.de (C.H.L.); jessica.bertrand@med.ovgu.de (J.B.); 2 Institute for Forensic Medicine, University Hospital Halle, 06120 Halle (Saale), Germany; kerstin.sitte@uk-halle.de; 3 Ludwig Boltzmann Institute of Osteology, Hanusch Hospital of OEGK and AUVA Trauma Centre Meidling, 1st Medical Department, Hanusch Hospital, 1140 Vienna, Austria

In the original publication [1], Andrea Schwab should have been listed as the corresponding author. Due to an oversight by the authors, Andrea Schwab was not included in this role. The current version has corrected this omission. The authors state that the scientific conclusions are unaffected. This correction was approved by the Academic Editor. The original publication has also been updated.

## References

[1] Schwab A., Jahn J., Sitte K., Lohmann C.H., Bertrand J., Gamsjaeger S. (2025). Spatial Raman Spectroscopy to Characterize (Sulfated) Glycosaminoglycans in Human Articular Cartilage. Int. J. Mol. Sci..

